# Transcriptome profiling of flax plants exposed to a low-frequency alternating electromagnetic field

**DOI:** 10.3389/fgene.2023.1205469

**Published:** 2023-06-07

**Authors:** Kamil Kostyn, Aleksandra Boba, Bartosz Kozak, Dariusz Sztafrowski, Jan Widuła, Jan Szopa, Marta Preisner

**Affiliations:** ^1^ Department of Genetics, Plant Breeding & Seed Production, Faculty of Life Sciences and Technology, Wroclaw University of Environmental and Life Sciences, Wroclaw, Poland; ^2^ Faculty of Electrical Engineering, Wroclaw University of Science and Technology, Wroclaw, Poland; ^3^ Faculty of Biotechnology, University of Wroclaw, Wroclaw, Poland

**Keywords:** electromagnetic field, flax, antioxidants, stress, transcriptome, RNA-seq

## Abstract

All living organisms on Earth evolved in the presence of an electromagnetic field (EMF), adapted to the environment of EMF, and even learned to utilize it for their purposes. However, during the last century, the Earth’s core lost its exclusivity, and many EMF sources appeared due to the development of electricity and electronics. Previous research suggested that the EMF led to changes in intercellular free radical homeostasis and further altered the expression of genes involved in plant response to environmental stresses, inorganic ion transport, and cell wall constituent biosynthesis. Later, CTCT sequence motifs in gene promoters were proposed to be responsible for the response to EMF. How these motifs or different mechanisms are involved in the plant reaction to external EMF remains unknown. Moreover, as many genes activated under EMF treatment do not have the CTCT repeats in their promoters, we aimed to determine the transcription profile of a plant exposed to an EMF and identify the genes that are directly involved in response to the treatment to find the common denominator of the observed changes in the plant transcriptome.

## Introduction

Plants have evolved in the presence of natural electromagnetic fields (EMFs) generated by Earth and its ionosphere, and they use these fields for important physiological processes such as photosynthesis and orientation (Yamashita et al., 2004; Tang et al., 2018). The EMF is a combination of electric and magnetic fields of force. Fundamentally, electromagnetic fields are created by particles with electric charge or magnetic moment. However, the field itself does not carry electric charge, magnetic moment, or mass (one electromagnetic field cannot directly interact with another electromagnetic field). Electromagnetic fields carry energy and momentum which they can give to particles with electric charge or magnetic moment (Baird, 2021), whereas the electric component results from the very existence of a charged particle, and the magnetic field requires it to be on the move. Electric fields are easily shielded or weakened by walls and other objects, whereas magnetic fields can pass through buildings, living things, and most other materials. The EMF produced by the Earth’s core steadily acts on living systems, although its intensity varies depending on the location. At the surface of Earth, the vertical component is maximal at the magnetic pole, which is approximately 67 μT, and is zero at the magnetic equator. The horizontal component is maximal at the magnetic equator, which is approximately 33 μT, and is zero at the magnetic poles (Kobayashi et al., 2004). However, with the advent of modern technology, plants are exposed to artificial electromagnetic fields generated by human-made devices such as power lines, cell phones, and Wi-Fi routers. The impact of these fields on plant growth and development has been the subject of much research in recent years. Although some studies suggest that electromagnetic fields can have negative effects on plant growth and reproduction, others indicate that they may have positive effects by stimulating cellular activity and increasing resistance to environmental stress (Maffei, 2014). In general, the EMF can be divided into constant (produced by direct currents) and variable (produced by alternating currents) fields. The alternating currents are rarely found in nature. The Earth’s core can be viewed as a gigantic dynamo, which reverses very slowly (183 times during the last 83 million years), although with variable rates (Banerjee, 2001), and thus can be considered a source of a constant magnetic field. There are two main categories of the alternating EMFs: high-frequency (HF-EMFs) and low- to-mid-frequency EMFs (with extremely low-frequency EMF (ELF-EMF) of 0–300 Hz).

The influence of anthropogenic electromagnetic fields on animals has been studied for quite a long time, and both beneficial and adverse effects on animals, depending on the frequency, intensity, and duration of exposure, as well as the specific characteristics of the animal and its environment, have been observed (Levitt et al., 2022). The first reports on the magnetosensitivity of plants appeared almost a century ago (Ssawostin, 1930). Since then, developmental studies on plant responses have been performed at various MF intensities and frequencies. In 1963, it was found that an MF of relatively low intensity could be effective in stimulating or initiating plant growth responses (Pittman, 1963). Similarly, sunflowers exposed to a 20-µT stationary magnetic field showed increased fresh weight (but not dry weight) of shoots and roots (Fischer et al., 2004). Low-frequency EMF exposure of wheat plants caused increased relative water content accompanied with a lower level of transpiration, elevated photosynthesis activity indexes, and increased leaf area. However, the effects were much more pronounced in the presence of an additional factor (drought) than without the stressor (Mshenskaya et al., 2023). A substantial number of studies on EMF effect on plants concern the effect of EMF on seed germination. In many cases, the pre-sowing exposure of seeds to EMF yielded a positive effect on seed germination. An ELF-EMF applied to dormant seeds of barley, corn, beans, wheat, certain tree fruits, and other tree species increased the subsequent seedling growth rate (Rochalska and Orzeszko-Rywka, 2005). A static MF of 87 × 10e3 to 226 × 10e3 μT intensity for 100 min improved the germination of mung bean seeds (Mahajan and Pandey, 2014). The biostimulation of the common bean seeds with the static EMF contributed to a higher germination capacity, energy, and strength (Pszczółkowski et al., 2023). ELF-EMF exposure (12 × 10e3 μT for 12 h) had a potentially positive effect on tobacco, which showed increased growth and chlorophyll content in *in vitro* culture (Riry et al., 2017). Seed germination energy and germination capacity of carrot (*Daucus carota*) were increased after alternating EMF exposure with the shielding of the electric component. On the other hand, onion seeds treated with EMF in the µT range led to significantly lower germination capacity and a higher percentage of abnormal deformed seedlings than untreated seeds (Górski et al., 2019). Furthermore, negative effects on onion, including reduced root growth, chromosomal aberrations, and genotoxic effects that increased with exposure duration, were observed after HF-EMF treatments (Chandel et al., 2019; Kumar et al., 2020). ELF-EMF induced oxidative damage and modified volatile components in pine needles (Esim et al., 2017). ELF-EMF treatment showed that the stimulatory effect of the electromagnetic field exposure on amaranth seeds tended to decrease as the duration of exposure increased, leading to adverse effects when the exposure time exceeded 30 min (Apasheva et al., 2006).

The aforementioned literature indicates that the results obtained after EMF treatment of plants differ and no clear conclusion can be drawn. It results probably from no standardized treatment procedures, but the studied plant species may be equally relevant. However, a few possible mechanisms behind the effects of EMF on living organisms have been proposed. Some reports indicate alterations in plasma membranes (Escobar et al., 2020) along with ROS metabolism activation after exposing plants to EMF (Zareh, 2015; Mshenskaya et al., 2022). The effects of the magnetic field on plants, fungi, and microbes can be elucidated by ion cyclotron resonance and the radical-pair model. These two mechanisms also play an essential role in the magneto-reception of organisms (Radhakrishnan, 2019). Cryptochromes are blue-light photoreceptor flavoproteins of size 50–70 kDa that regulate a variety of processes in organisms ranging from bacteria to humans. Enhanced cryptochrome-mediated inhibition of hypocotyl (stem) elongation in *Arabidopsis thaliana* in a 500-μT magnetic field, relative to the controls in the ambient magnetic field (approximately 40 μT), was observed under blue-light irradiation but neither under red light (where the mediating photoreceptors are phytochromes) nor in total darkness. This was interpreted in terms of a flavin–tryptophan radical pair formed by photoinduced electron transfer within a cryptochrome photoreceptor (Giovani et al., 2003; Ahmad et al., 2007). In fact, a plethora of studies on the possible mechanism of EMF action suggest that free radicals are the key factors directly produced due to exposure (Portaccio et al., 2005; Kivrak et al., 2017; Lai, 2019; Gao et al., 2021). This reprograms the enzymatic activity, transport of the metabolites including growth regulators, and also the transport of charged solutes possibly through the “Hall” effect for plant growth improvement. In addition, the critical role of calcium, a crucial second messenger in plants, has long been pointed out. The importance of calcium in the establishment of the plant response is also highlighted by the fact that early gene expression associated with EMF exposure involves at least two calcium-related products (calmodulin and calcium-dependent protein kinase) (Pazur and Rassadina, 2009).

Our world changed undisputedly with the development of electricity. In both the industry and our houses, circuits are supplied with alternating current (AC) of 50 Hz frequency (60 Hz in some countries). The frequency of 50 Hz has become an integral part of the fabric of modern society, providing a consistent and reliable source of electricity to millions of people every day, rendering the exposures to ELF-EMF ubiquitously. The use of electricity has necessitated the creation of a huge power grid entwining almost every corner of the surface that our civilization occupies. Electricity is mainly transmitted through overhead power lines. They are cheaper and easier to maintain than the underground grid, but, on the other hand, are a source of ELF-EMF that can influence everything around them, including vast areas of cultivation. A typical high-voltage power line (110 kV) has several hundreds of amps passing through it. Assuming that the typical height of a power pylon is ca. 6 m and the current amperage is 500 amps, magnetic induction can be calculated according to the modified Ampere’s law as follows:

$$B=\frac{\mu_{0}I}{2\pi R},$$

where B is the magnetic induction, μ_0_ is the magnetic constant (permeability of free space), I is the electric current, and R is the radial distance away from the wire and equals 16,67 μT. The value may not seem significantly high, but considering the continuous nature of the exposure during the growth of crops in the vicinity, the influence should be considered relevant.

Overall, the influence of electromagnetic fields on plants remains an important area of investigation as it has implications for both agriculture and the environment. In our study, we aimed to investigate the effect of ELF-EMF (500 μT, 50 Hz) on flax. Flax (*Linum usitatissimum* L.) is a plant with a rich history of cultivation dating back to ancient times. It is grown for both its seeds, which are rich in oil and fiber, and its blue flowers, which are used for ornamental purposes. Flax is an important crop in agriculture due to its versatility and numerous health benefits. Flax seeds are a rich source of omega-3 fatty acids, lignans, and other nutrients, making them a popular superfood. Flax fiber, on the other hand, is used to produce linen fabric, paper, and other products. Flax oil is also used in various industrial applications, including paint, varnish, and soap. In addition to its economic importance, flax also plays a vital role in sustainable agriculture. It is a hardy crop that can be grown in a variety of soil types and climates without the need for synthetic fertilizers or pesticides. Flax also has a deep root system that helps prevent soil erosion and improve soil health. However, flax is constantly exposed to various threats, with water deprivation and pathogen infections in the first place. Our preliminary data showed increased ROS levels and changes in the expression of genes involved in ROS processing after EMF treatment. This prompted us to investigate the effects of ELF-EMF on flax transcript levels by RNA sequencing (NGS). This could provide new insights into the molecular mechanisms of flax triggered by the ELF-EMF.

As mentioned previously, numerous studies focus on seeds as the subject of EMF treatments and aim to improve the germination characteristics. From this perspective, it would be interesting to investigate the mechanisms triggered by EMF. On the other hand, the transcriptional machinery of seeds, especially during dormancy, is reduced, and genes that are transcriptionally active potentially regulate progress toward germination (Cadman et al., 2006; Venglat et al., 2011). We chose flax seedlings because during the seedling stage, the plant’s ability to cope with environmental stresses has the highest impact on its further development or even survival. Moreover, at this stage of development, the plant transcriptional activity is not as biased as in the seeds; thus, investigating the molecular mechanisms triggered by the ELF-EMF should be easier to interpret.

## Materials and methods

### Plant growth conditions and EMF treatment

Flax seeds (*L. usitatissimum*, var. Nike) obtained from the Flax and Hemp Collection of the Institute of Natural Fibers and Medicinal Plants in Poland were sterilized in 50% PPM solution (Plant Cell Technology, Washington, DC, United States) for 10 min and rinsed in sterile water three times. Then, they were placed on Murashige and Skoog (MS) medium solidified with 0.9% agar and grown in a phytotron (16 h under the light intensity of 100 μmol m^−2^s^−1^, 60% relative humidity, and temperature of 21°C, and 8 h in darkness at 16°C and relative humidity of 70%). After 14 days, the seedlings were exposed to a 50 Hz, 500 μT alternating electromagnetic field (EMF) for 30 min under the aforementioned phytotron light conditions. No significant effect of the environment on the experimental results with the applied EMF was observed.

**FIGURE 1:**
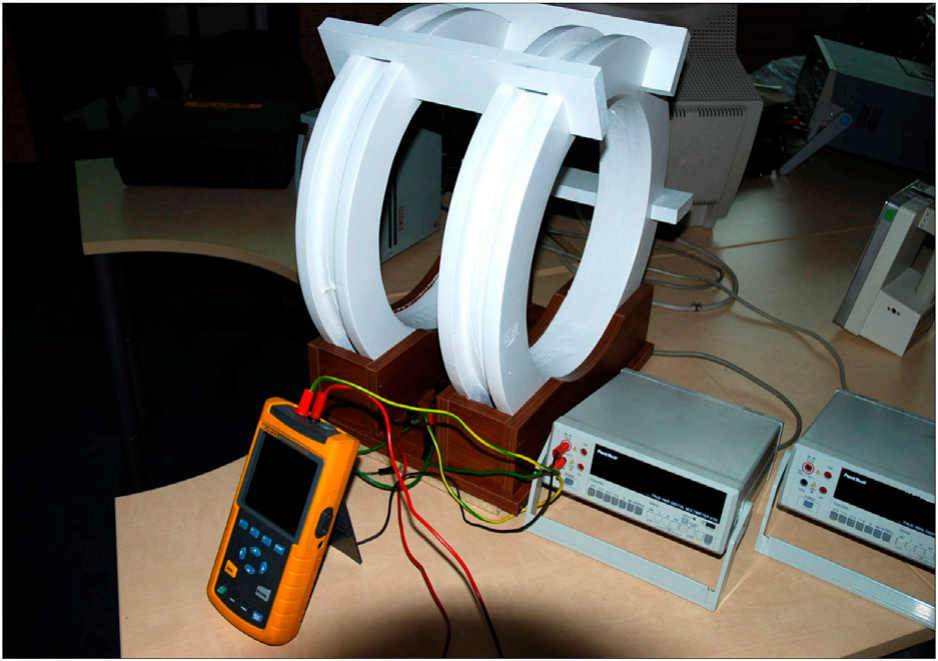
Scheme of the ELF-EMF exposure system.

The EMF exposure system used in the experiment consisted of two identical Helmholtz coils positioned vertically at a distance of 216 mm to ensure horizontal orientation of the magnetic flux. The coil inner and outer diameters were 400 mm and 462 mm, respectively, and the width was 40 mm. The Helmholtz coils were made of a 2.12-mm-thick copper wire wound 225 times (Figure 1; Supplementary Figure S2). The output of the autotransformer (ZWE Eltra, Bydgoszcz, Poland) connected to the electric energy supply was an electric source for a sinusoidal 50-Hz alternating current (sinusoidal 50-Hz MF) in the experiment. The current intensity was controlled by the ammeter (Multimeter Fluke 8846A; Fluke, Cleveland, OH, United States). The MF strength in the center of the Helmholtz coils can be calculated using the following formula derived from Biot–Savart’s law:

$$H_{calculated}=0.7155∙\frac{N∙I}{r}=0.7155∙\frac{225∙I}{0.212}=759∙I,$$

where I is the value of current, r is the radius of coil wires, and N is the number of windings.

Higher harmonic waves in the current were monitored during the experiments (Fluke 43 Power Quality Analyzer, Fluke, Cleveland, OH, United States) and did not exceed 2%. Magnetic induction was verified using the ESM-100 3D H/E Field Meter (Maschek, Germany).

The exposure was carried out on 14-day-old flax seedlings placed in Petri dishes on MS medium in the geometrical center of Helmholtz coils. After the EMF exposure, the plants were grown for 12 h, and then, the seedlings were collected, immediately frozen in liquid nitrogen, and stored at −80°C for further analyses.

### RNA isolation and sequencing

Extraction of total RNA from the flax seedling tissue ground in liquid nitrogen was performed using the mirVana™ miRNA Isolation Kit (Thermo Fisher) according to the manufacturer’s protocol. Genomic DNA was removed using DNase I (Thermo Fisher). The Agilent 2100 Bioanalyzer (Agilent RNA 6000 Nano Kit) was used for RNA quality assessment. RNA samples with a RIN value >7.5 were used for RNA-seq library construction. mRNA was isolated from the total RNA by using the oligo (dT) method. After fragmentation, it served for the first cDNA strand synthesis. Synthesis of the second cDNA strand was followed by adapter joining. The cDNA fragments of suitable size were amplified with PCR and sequenced on an Illumina HiSeq 2500 device. In order to filter the low-quality readings, reads with adapters, and reads with unknown bases (N bases >5%), Trimmomatic software was used (Bolger et al., 2014). The clean reads were aligned to the reference genome (Wang et al., 2012) using HISAT2 software (v2.1; https://daehwankimlab.github.io/hisat2/) with the following parameters: “-q—phred64—dpad 0—gbar 99999999—mp 1,1—np 1—n-ceil L, 0,0.15—no-mixed—no-discordant -p 38 -k 10” [https://doi.org/10.1038/nprot.2016.095]. The gene expression level was calculated using featureCounts software in the Subread package (v1.6; http://subread.sourceforge.net/) (Liao et al., 2013). The analysis pipeline is given in Supplementary Figure S1.

### Detection of DEGs and GO analysis

Differentially expressed genes (DEGs) between samples were identified using DESeq2 [https://doi.org/10.1186/s13059-014-0550-8]. The parameters used were as follows: “Fold Change > = 2 and Adjusted *p*-value < = 0.001.” As some transcripts identified against the reference genome had no GO number assigned, we searched the genome database available for black cottonwood (*Populus trichocarpa*) (Torr. & Gray) using the BLASTx algorithm. The results were filtered with the E-value threshold equal to or less than 1e-40. DEGs were classified based on the GO annotation results and reference genome annotation. GO functional enrichment using the goseq package in R [https://doi.org/10.1371/journal.pone.0246052] was also performed. The false discovery rate (FDR) for each *p*-value was calculated, and terms with FDR <0.01 were considered significant for our analysis (Love et al., 2014).

### Reverse transcription real-time PCR

Transcript levels were determined using RT-qPCR using the StepOnePlus™ Real-Time OCR System (Applied Biosystems, United States) on the cDNA matrix in three independent repeats. Primers used in the reactions were designed in LightCycler^®^ Probe Design v2 (Roche, Switzerland) software (Supplementary Table S1). For the reactions, the DyNAmo SYBR Green qPCR Kit (Thermo Fisher Scientific, United States) was used. cDNA was dissolved five times prior to the experiment. The annealing temperature was 57°C. Changes in the transcript levels of the examined genes were presented as relative quantification with regard to the control. *Actin* gene was used for reference.

### Determination of antioxidant capacity

Frozen flax seedling tissue (100 mg) was ground in liquid nitrogen and extracted to methanol containing 1% HCl in an ultrasonic bath for 10 min and centrifuged at 12,000 *g*, 4°C for 10 min (three times to 1 mL). The collected supernatants were combined, dried under nitrogen flow, and re-suspended in 200 μL of methanol. The reaction with 2,2-diphenyl-1-picrylhydrazyl (DPPH) was used to determine the antioxidant capacity of the extracts.

In the DPPH assay, 6 μL of plant extract was added to 200 μL of 0.1 mM DPPH. Following incubation (15 min at room temperature), absorbance at *λ* = 515 nm was measured. For the control, 200 μL of DPPH solution with 6 μL of methanol was used. Pure methanol was used as the blank sample. The results were expressed as equivalents of trolox antioxidant potential (inhibition of the free-radical reaction expressed as a percentage) calculated according to the following equation:

$$P=1-\frac{A_{s}}{A_{c}}∙100\%,$$

where P is the antioxidant potential, A_s_ is the absorbance of the examined sample solution, and A_c_ is the absorbance of the DPPH radical solution (Brand-Williams et al., 1995).

### Infection assay

In this experiment, 2-week-old flax plants grown in sterilized vermiculite with MS medium (35 mL MS per 50 g vermiculite) were exposed to a 50 Hz, 500 μT alternating electromagnetic field (EMF) for 30 min. Immediately after exposure, they were infected with 500 ul of 10-e5 spores of *Fusarium oxysporum* freshly prepared as follows: *F. oxysporum* grown on solid PDA medium at 28°C for 2 weeks were rinsed with 2 mL of sterile water, which was then filtered through sterile gauze and moved to an Eppendorf probe. The *Fusarium* spore count in the suspension was determined under a microscope in the Thoma cell counting chamber. After 10 days of growth, the plants were collected, and the presence of fungal DNA was assayed using the RT-qPCR method with primers specific to fungal murein transglycosylase gene. Pure water was used in the control.

### Dual staining of flax plants treated with ELF-EMF and infected with *F. oxysporum*

The plants (exposed and not exposed (control) to ELF-EMF and then infected with *F. oxysporum*) were collected and subjected to clearing with 0.15% TCA in an ethanol: chloroform mixture (4:1; v/v) for 48 h, divided into green parts and roots, and then submitted to staining with safranin and solophenyl flavine 7GFE (0.1% w/v in 0.1 M Tris/HCl, pH 8.5) as follows: the plants were submerged in safranin solution (0.2% w/v safranin in 10% v/v ethanol) for 5 min and then washed three times with water. Subsequently, the plants were stained for 10 min with solophenyl flavine 7GFE (0.1% w/v in 0.1 M Tris/HCl, pH 8.5) and washed again with water (four times) (Knight and Sutherland, 2011). Roots and green parts were separated and mounted on slides, observed under the epi-fluorescence microscope Olympus BX50 using UV excitation light (360–370 nm), and documented using the Olympus DP71 camera and Cell^B software (Olympus Optical Co.). Images, taken at different depths, were processed using Helicon Focus 6.5.2 Pro (Helicon Soft Ltd.) and CorelDRAW 2017 (Corel Corporation). False colors (cyan) were assigned to the *F. oxysporum* cells.

### Identification of promoter elements

Significantly enriched promoter motifs were identified separately for statistically significant up- and downregulated genes using HOMER (v4.9, Hypergeometric Optimization of Motif EnRichment, http://homer.ucsd.edu/homer/motif/) with the following parameters: motif length of 8, 10, or 12 bp within 500 bp upstream or downstream from the transcription start site. Motif enrichment analyses were performed relative to the background gene set of all expressed genes.

### Statistical analysis

The raw count matrix generated using featureCounts was normalized with rlog function in the DESeq2 package. Next, these data were used in principal component analysis (PCA). Calculation was performed in R software with prcomp function and visualized using the FactoMineR (Lê et al., 2008) and ggplot2 packages (Wickham, 2009).

All experiments were performed using at least three biological repeats. The results were presented as the mean values ± standard deviation. To assess the significance of changes, one-way ANOVA with Tukey’s *post hoc* test or the Kruskal–Wallis test was used (Statistica, Dell, Round Rock, TX, United States).

## Results and discussion

### Influence of EMF on the H_2_O_2_ level and the antioxidant potential in flax seedlings

Electromagnetic fields can be considered an abiotic stressor, which induces the alteration in the chemical content of plant cells by oxidative stress (Shabrangy and Majd, 2018). Under a stress stimulus, a plant produces reactive oxygen species (ROS) through several biochemical transformations. ROS include hydroxyl radicals (^•^OH), superoxide anions (O_2_^•−^), hydrogen peroxide (H_2_O_2_), and singlet oxygen (^1^O_2_) (Mittler et al., 2004). A superoxide anion radical (O_2_^•−^) is usually the first ROS formed by the enzymatic process, autooxidation reaction, and nonenzymatic electron transfer reactions, in which an electron is transferred to molecular oxygen. O_2_^•−^ can be generated in the organelles, where the electron transport chains occur (plastids and mitochondria), and membrane-dependent NADPH oxidase (respiratory burst oxidase homolog (RBOH) proteins) systems. The superoxide anion (O_2_^•−^) is the precursor of various ROS because of its instability (a relatively short half-life of 2–4 μs) and strong oxidation/reducibility. The superoxide anion may serve as a substrate for superoxide dismutase (SOD), which catalyzes its dismutation to H_2_O_2_. Hydrogen peroxide is considered an important redox molecule, given its specific physical and chemical properties, including remarkable stability within cells (half-life of 10^−3^ s) (Mhamdi and Van Breusegem, 2018). H_2_O_2_ is an important signaling molecule (Nazir et al., 2020) and thus must be strictly controlled under physiological conditions. This can be realized by its conversion to water and oxygen driven by catalase (CAT).

**FIGURE 2:**
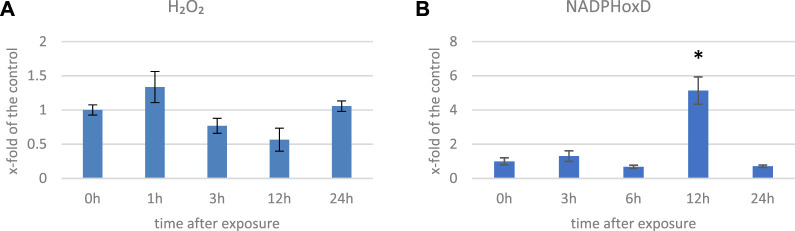
Level of H_2_O_2_ **(A)** and transcript level of NADPH oxidase (NadphoxD) **(B)** measured in several time points in flax exposed to the 500 μT, 50 Hz EMF. The bars represent the mean values of three biological repeats ± standard deviation. The asterisks mark statistically significant changes in relation to the control (*p* < 0,05).

We measured the H_2_O_2_ levels in several time points in flax exposed to 500 μT, 50 Hz EMF. After an initial increase (by 33% 1 h after exposure (hae)), the level of H_2_O_2_ decreased and reached 57% of the control at 12 hae and then increased at 24 hae (Figure 2A). The decrease in the H_2_O_2_ level was also observed in *Anthemis gilanica* seedlings, although due to a different experimental setup, the measurement was performed at a later time after the exposure (Hassanpour et al., 2021). NADPH oxidase is a membrane-bound enzyme with channel voltage-gating-associated ROS production which is known to be increased by EMF (Friedman et al., 2007). Although it is thought to be indirectly affected by the EMF (Georgiou and Margaritis, 2021), an increase in the transcript level of the corresponding gene (over three times more than the control) was observed at 12 hae (Figure 2B).

**FIGURE 3:**
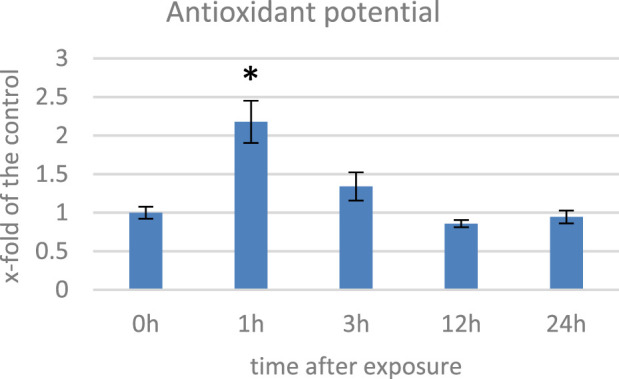
Antioxidant potential measured with the DPPH method in several time points in flax exposed to the 500 μT, 50 Hz EMF. The bars represent the mean value of three biological repeats ± standard deviation. The asterisks mark statistically significant changes in relation to the control (*p* < 0,05).

ROS in plants can damage cellular components and lead to cell death; thus, in response to oxidative stress, plants have an evolved complex antioxidant defense system, which includes enzymatic (e.g., catalase, peroxidase, and superoxide dismutase) and non-enzymatic antioxidants with polyphenols, terpenoids, and vitamins that work together to scavenge ROS and prevent cellular damage (Xu et al., 2017). We measured the antioxidant capacity of EMF-exposed flax plants’ methanol extracts and observed an increase of over twofold at 1 hae and its gradual decrease in the subsequent time points (Figure 3). Changes in the antioxidant potential correlated well with the changes in the level of oxygen peroxide. When in contact with a stressor, plants develop the early phase (2–6 h) of the stress response (Rapala-Kozik et al., 2012). During this time, ROS are generated in excess. To cope with them, activities of numerous genes are modulated, but before and during this process, the antioxidants already present in the cytoplasm provide the first line of defense. Under normal conditions, small molecular antioxidants like phenolic derivatives (including flavonoids) are present in the cell in the form of glucoside conjugates. Such modification usually decreases their activity but improves solubility in the cytoplasm, thus allowing for their widespread presence in the cell. When stress occurs, recruitment of these dormant ROS scavengers are activated through cleaving the glycosyl group (Brunetti et al., 2013; Behr et al., 2020). Although the time required for genetic expression depends on many factors, such as the type of stimulus and promoter activity, it usually takes hours until a steady-state expression state is achieved (Waters et al., 2017). Considering this, although the 1 hae time point is characterized with greater antioxidant potential, based on the expression of the NADPH oxidase gene, we selected 12 hae as the optimum time point for further analyses.

### Transcriptome sequencing and GO analysis

**FIGURE 4:**
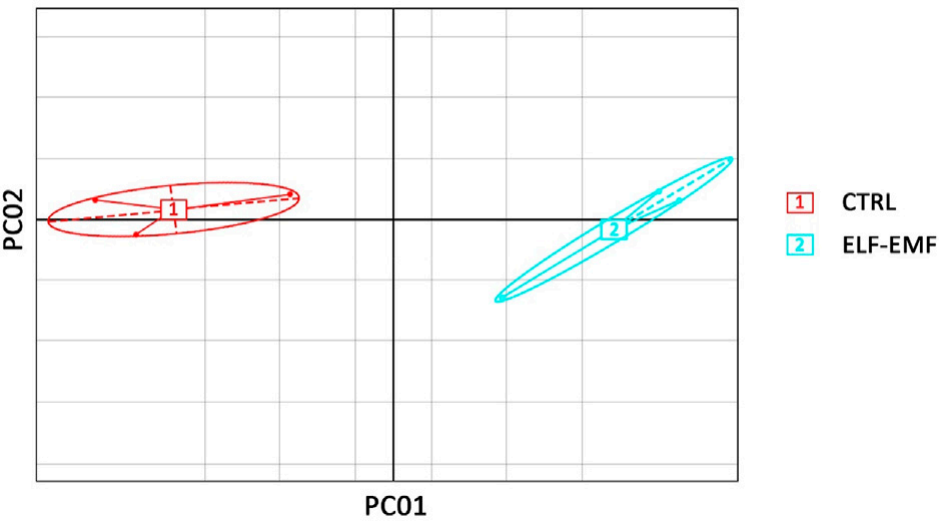
Principal component analysis on transcriptome data from flax seedlings exposed to the 50 Hz 500 μT ELF-EMF (2) and the control (1).

Transcriptomes of flax (*L. usitatissimum* L. var. Nike) were sequenced in the seedlings exposed to the 50 Hz, 500 μT ELF-EMF for 30 min and collected at 12 hae compared to the non-treated control. The main purpose of the study was the evaluation of changes (≥2-fold) in the transcript levels. Principal component analysis (PCA) was performed with PCA function in the FactoMineR (Lê et al., 2008) package of R software and visualized using the ggplot2 (Wickham, 2009) package of R software. The PCA results revealed good separation between the ELF-EMF-exposed and control samples (Figure 4) in the PC01 component.

**FIGURE 5:**
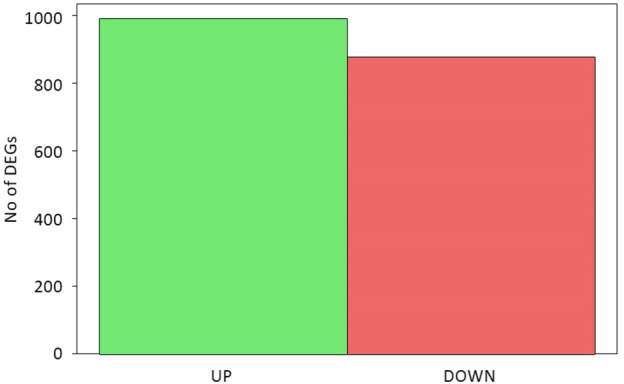
Statistics of differentially expressed genes.

**FIGURE 6:**
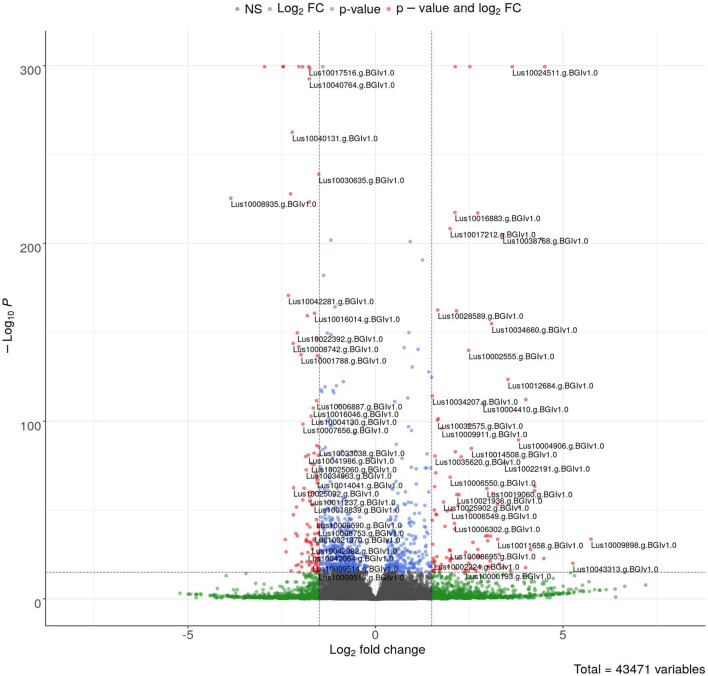
Volcano plot of DEGs. The colored points represent differentially expressed genes with alpha = 0.05 and log_2_FC = 1 (blue: downregulated and red: upregulated). The top 100 up- and downregulated genes are labeled.

The sequencing data were used for differentially expressed gene (DEG) analysis. The results are given in Supplementary Table S2. Among the 1,882 identified DEGs, almost 1000 were upregulated, while over 800 were downregulated (Figure 5). For quick visual identification of genes with large fold changes, a volcano plot is given in Figure 6. Then, Gene Ontology (GO) analysis was performed on differentially expressed genes.

**FIGURE 7:**
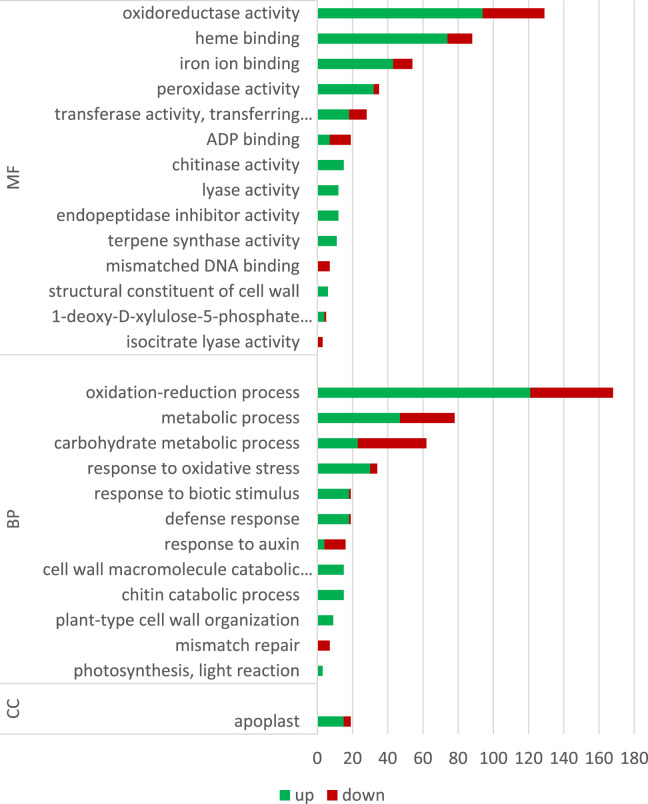
Gene Ontology (GO) classification.

GO analysis is typically performed after transcriptome sequencing to gain insights into the biological processes, molecular functions, and cellular components associated with the DEGs. By using GO analysis, genes can be annotated based on their functions and categorized into functional groups to better understand the biological mechanisms that underlie the observed differences in gene expression between the conditions being compared, the influence of ELF-EMF in this case. The number of DEGs of GO terms (categories) that were statistically and significantly overrepresented is given in Figure 7; Supplementary Table S3. The most upregulated genes involved in redox processes, response to oxidative stress, and heme binding/iron binding were identified. These results confirm previous findings on the influence of EMF on plants. For instance, among 34 peroxidase or peroxidase-related genes, only four were downregulated. Xiao-ju and Guo (1999) found an increase in the activity of catalase and peroxidase enzymes in tomato seeds pretreated with MF. An increase in catalase activity after a 30-min exposure of wheat plants to a magnetic field with frequencies of 14.3 Hz and 20.8 Hz by 60% and 20% relative to the control level was shown by Mshenskaya et al. (2022). In addition, all the three enzyme gene activities were shown to be increased after EMF treatment in the study conducted by Hassanpour et al. (2021). The authors showed increased levels of phenolic derivatives, which in turn translated into a higher antioxidant potential measured by the DPPH method. On the other hand, Alemán et al. (2014) showed decreased levels of SOD, CAT, and Apx activities in different developmental stages of coffee (except CAT in 4-month-old seedlings) after EMF treatment. The field intensity was 2 × 10e3 μT, but the exposure was only 3 min (4 times and 10 times shorter than those used in our experiments, respectively). Esim et al. (2017) ascribed adverse effects to the ELF-EMF by showing increased H_2_O_2_ levels and decreased antioxidant enzymes.

**FIGURE 8:**
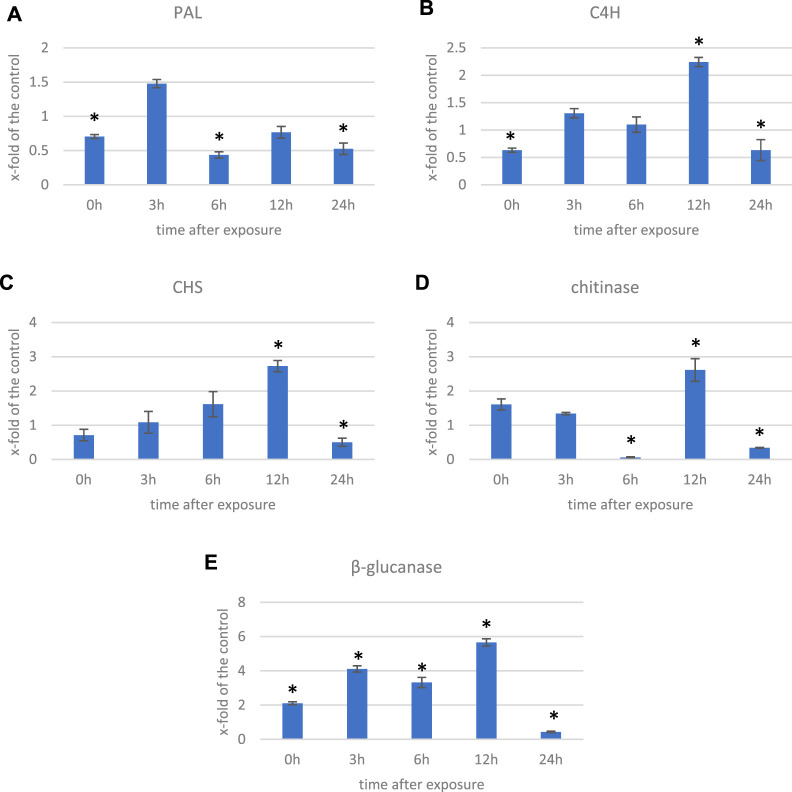
Transcript level of **(A)** phenyalanine ammonia lyase (PAL), **(B)** cinnamate-4-hydroxylase (C4H), **(C)** chalcone synthase (CHS), **(D)** chitinase, and **(E)** β-glucanase genes measured in several time points in flax exposed to the 0.500 μT, 50 Hz EMF. The bars represent the mean values of three biological repeats ± standard deviation. The asterisks mark statistically significant changes in relation to the control (*p* < 0.05).

**FIGURE 9:**
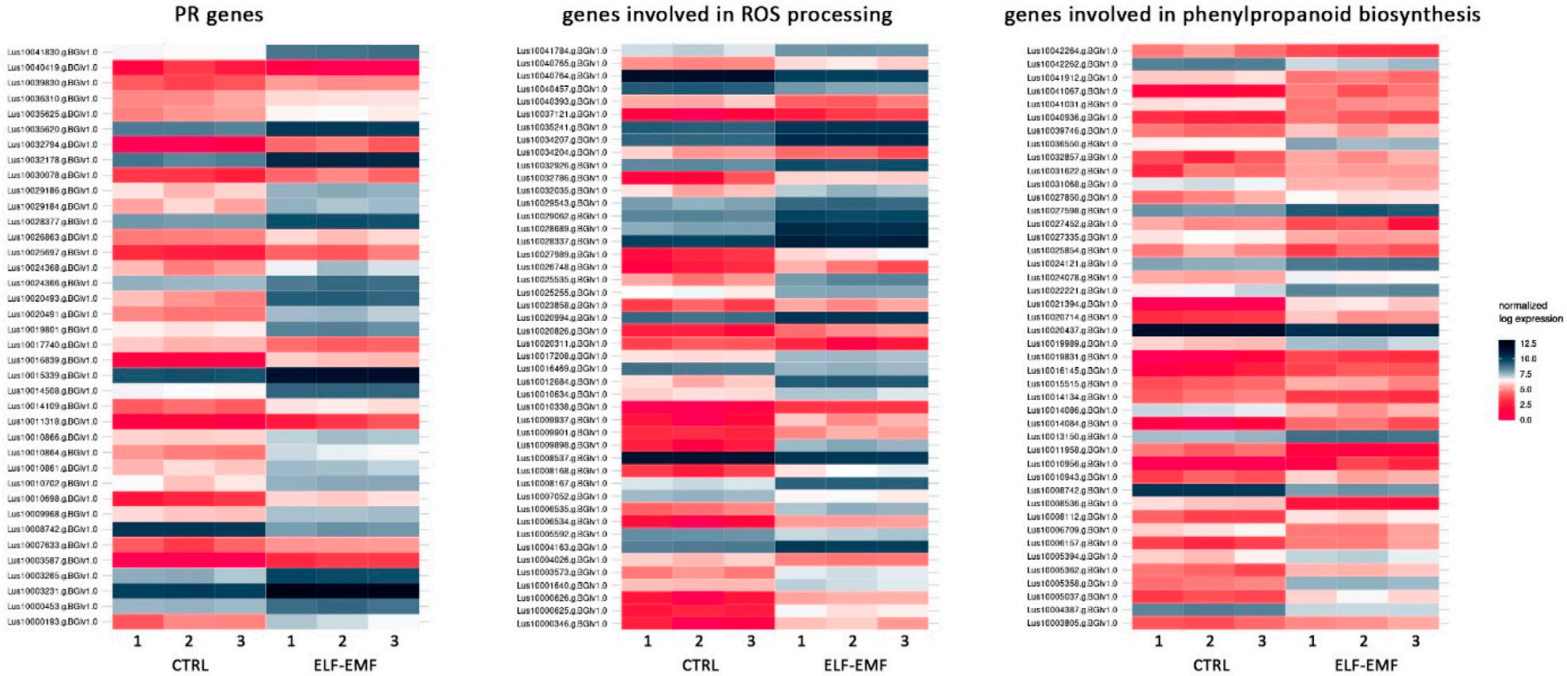
Heatmaps of the expression of PR genes, genes involved in ROS processing, and genes involved in phenylpropanoid biosynthesis in 4-week-old flax seedlings exposed to the 50 Hz, 500 μT ELF-EMF. The expression was normalized using the DESeq2 method.

In addition to the genes encoding enzymes directly involved in the antioxidant response to ROS, genes involved in low-molecular-weight antioxidants are important for the quenching of free radicals. Surprisingly, as phenylalanine ammonia lyase (PAL) gene expression is very sensitive to various stresses (Gholizadeh and Kohnehrouz, 2010; Jóźwiak-Żurek et al., 2011; Zhan et al., 2022), no *PAL* gene was found to be considerably (over 2-fold) changed after ELF-EMF exposure, while the activity of PAL, the key enzyme in phenylpropanoid biosynthesis (Kostyn et al., 2012), as well as its encoding gene expression, was shown to be significantly increased in tobacco after EMF treatment (Radmard Titkanlo et al., 2018). We verified the expression of *pal* gene using RT-qPCR, and its transcript level was generally downregulated in all time points. There was an increase only at 3 hae in the transcript level, reaching 1.48-fold of the control (Figure 8A). However, the expression of the gene encoding the subsequent enzyme cinnamate-4-hydroxylase (C4H), which oxidizes cinnamic acid to 4-coumaric acid, was over 2.5 times higher after ELF-EMF exposure than the control, which was verified using RT-qPCR (Figure 8B). Its expression was the highest at 12 hae and reached 2.24-fold of the control. Moreover, expression of the gene encoding 4-coumarate-CoA ligase (4CL), which converts 4-coumaric acid to its more active form of 4-coumaroyl-CoA, was over 34 times higher. 4-Coumaroyl-CoA is a starting point for the synthesis of a whole gamut of phenolic derivatives, including phenolic acids (caffeic acid, ferulic acid, lignin, lignans, flavonoids, and anthocyanins). It is noteworthy that all the aforementioned three genes are activated by endogenous H_2_O_2_ (Cheng et al., 2019). No significant changes were observed for the genes involved in lignin biosynthesis (like the two most important cinnamoyl-CoA reductase (CCR) and cinnamyl alcohol dehydrogenase (CAD)). The level of lignin in such young seedlings, especially grown under *in vitro* conditions, was below the detection limit of the “acetyl bromide” method (Hatfield et al., 1999) (data not shown). On the other hand, the plant’s reaction to oxidative stress, as during infection, can be manifested by enhanced polymerization of cell wall components, including lignin. The resulting tightening of lignin proceeds mainly via “end-wise” polymerization, in which an oxidized monolignol radical cross-couples with a radical formed on the free-phenolic ends of a growing lignin polymer. The ROS necessary for this process is produced by NADPH oxidases and laccases (Cheng et al., 2019; Lee et al., 2019; Tobimatsu and Schuetz, 2019). Our transcriptome analysis showed that both the NADPH oxidase and laccases were upregulated after the exposure to ELF-EMF. Among other polyphenol derivatives with antioxidant potential are flavonoids and anthocyanins. Transcriptome analysis revealed that the expression of chalcone synthase (*chs*) encoding the key enzyme in flavonoid biosynthesis was over two times higher than that in the non-treated control, which was verified using RT-qPCR (Figure 8C). Flavonol synthase (*fls*) gene transcription was also increased (4.7 times higher) compared to the control. The increase in the transcript level of flavonoid 3′-hydroxylase gene (*f3′h*) but not flavonoid 3′,5′-hydroxylase was also noticed, suggesting the redirection of the pathway to quercetin production. The level of dihydroflavonol reductase (*dfr*) gene transcript was downregulated after exposure to ELF-EMF (0.4-fold of the control). This, together with the overexpression of anthocyanidin synthase (*ans*) and anthocyanidin glucosyltransferase (*ugt*) genes, suggests that anthocyanin synthesis from leucoanthocyanidins is preferred. The phenylpropanoid biosynthesis pathway also includes the production route of salicylic acid (SA), a known phytohormone participating in the plant’s response to stress. SA levels were shown to increase after the use of the pulsed magnetic field on raspberry plants (Upadyshev et al., 2021). This phytohormone is known to participate in the activation of many genes involved in the plant’s response to stress, including pathogen attack. Moreover, the methylated derivative of SA (methyl salicylate (MeSA)) is volatile and shown to act as an inducer of plant defense mechanism against pathogens and certain herbivores (Boba et al., 2017; Ninkovic et al., 2021). The methylation of SA is catalyzed by benzoic acid/salicylic acid methyltransferase (BSMT). In our experiment, the *BSMT* gene transcript level was found to be increased in flax seedlings exposed to ELF-EMF (almost 2.4 times higher than the control). At the same time, the transcript level of salicylic acid glucosyltransferase (SAGT) was decreased. Data on the expression of the PR genes, genes involved in ROS processing, and phenylpropanoid biosynthesis are given in Figure 9 in the form of heatmaps.

All of the aforementioned observations resemble the plant’s response to pathogen attack, and many genes that are activated upon infection are observed to be upregulated also due to the ELF-EMF exposure. Moreover, we found that the number of DEGs of the WRKY transcription factors (TFs) was higher than the control. WRKY transcription factors, mainly induced by SA upon infection, act in a complex defense response network as both positive and negative regulators (Pandey and Somssich, 2009). In addition, due to infection, pathogenesis-related proteins (PRs) are biosynthesized. Among all PR genes identified in the transcriptome of flax exposed to ELF-EMF, the majority was upregulated. For instance, chitinase transcript levels were higher (2- to 57.5-fold) than those of the control. Similarly, β-glucanase transcripts were more abundant in the ELF-EMF-exposed flax seedlings than in the control. Both chitinase and β-glucanase genes are activated in flax under pathogen attack (Wojtasik et al., 2019). Their expression levels were confirmed by RT-qPCR (Figures 8D,E).

### Influence of ELF-EMF exposure on infection susceptibility of flax seedlings

**FIGURE 10:**
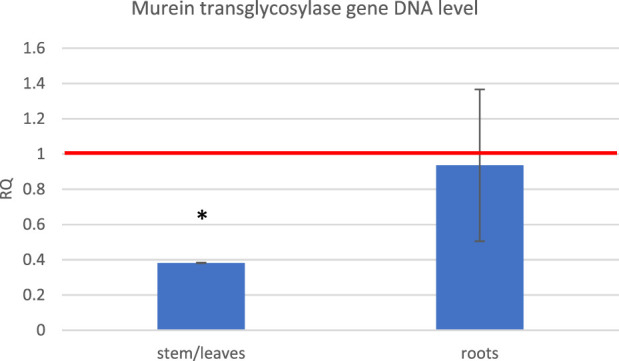
Level of the fungal murein transglycosylase gene DNA measured in the green parts and roots of flax seedlings exposed to 500 μT, 50 Hz EMF compared to the non-treated (only infected) control (red horizontal line (RQ = 1)). The bars represent the mean value of three biological repeats ± standard deviation. The asterisks mark statistically significant changes in relation to the control (*p* < 0.05).

Based on the observation that exposure to the ELF-EMF leads to the upregulation of genes usually activated upon pathogen infection, we decided to perform infection tests on flax seedlings exposed to the ELF-EMF compared to the untreated control. The seedlings were infected with *F. oxysporum* spores directly after exposure. After 10 days, we measured the level of fungal murein transglycosylase gene by RT-qPCR as a marker of the fungal cells already present in the plant. We divided the plant material into the green parts (stems/leaves) and roots. Although no major changes were observed in the roots, the number of *F. oxysporum* cells in the green parts was significantly lower (Figure 10).

**FIGURE 11:**
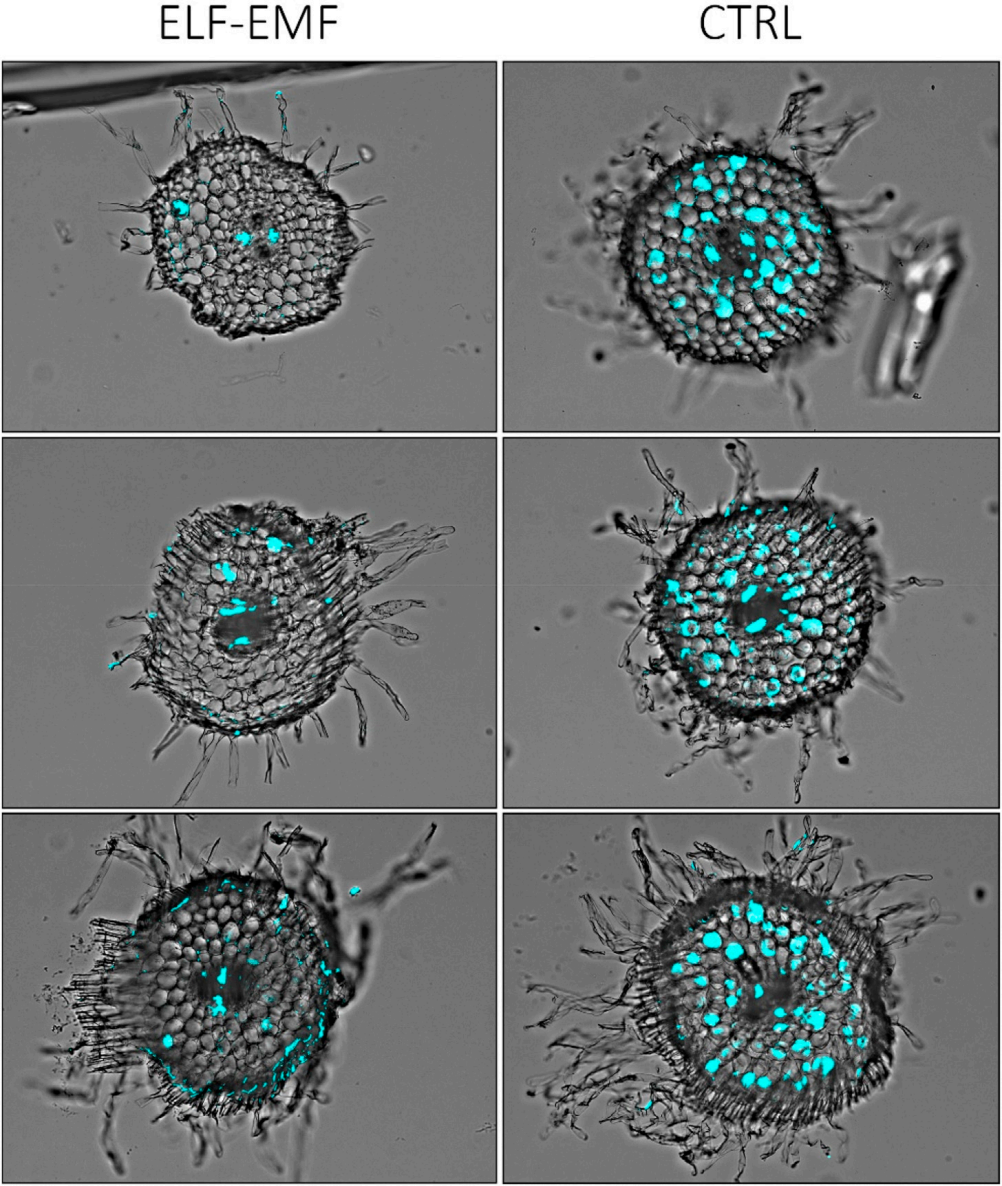
Microscopic image of stem sections of 2-week-old flax seedlings treated with ELF-EMF and non-treated control (CTRL) infected with *F. oxysporum*. The fungal cells in the vascular bundles are false-colored (cyan) using Cell^B software (Olympus Optical Co.).

To visually confirm that there is less fungal penetration into the green parts of flax seedlings treated with the ELF-EMF before infection, we performed histochemical staining of the fungus and observed the stem sections under the microscope. Solophenyl flavine staining clearly showed a less number of *F. oxysporum* cells in the ELF-EMF-exposed stem tissue than in the control (Figure 11).

## Conclusion

We realize that the selected 12 hae time point may be too late for the investigation of the mechanisms of EMF action in plants or direct influence of EMF on the regulation of gene expression. On the other hand, experiments conducted at 12 hae allowed us to observe some effects, most probably of secondary nature, that were produced in flax plants and resemble the plant’s response to pathogen infection. ROS, being produced due to EMF action in living organisms, is the probable trigger for the observed changes in the exposed flax plants. It is well known that ROS is produced upon infection in the so-called oxidative burst, which is the natural plant’s early response to pathogen attack. ROS is also a known signaling molecule which, among others, induces the expression of defense-related genes (Khan et al., 2023). Among the early responses of flax plants are phenylpropanoid production (Kostyn et al., 2012) and methyl salicylate release (Boba et al., 2017), and genes involved in these processes were activated after ELF-EMF exposure of flax plants. Similar to that in nature, in our experiment, the infection of flax seedlings proceeded through the roots. After the initial penetration of the root tissue by *F. oxysporum*, the pathogen grows through the vascular bundles to infect the whole plant. Based on our infection experiments, we concluded that the ELF-EMF exposure hindered the growth of the fungus hyphae. This effect may result from both the reaction of the plant altered by the EMF and from the very influence of EMF on *F. oxysporum*; in particular, there are reports on the inhibition of fungal growth by the electromagnetic field (Nagy and Fischl, 2004; Gorczyca et al., 2011). Finding the exact location where the fungal growth inhibition takes place (roots or stem) requires additional studies on root transcriptome changes after ELF-EMF exposure and *F. oxysporum* infection. Taking all these into account, the EMF exposure may be considered a priming method, which can prepare plants for upcoming infection, as shown in our study.

Based on the RNA-seq results presented in our study, we determined the known and plausible promoter elements of the genes statistically significantly up- and downregulated upon EMF exposure (Supplementary Table S4). Our next step is the *in silico* analysis of the promoter elements in order to identify possible EMF response elements (EMREs), paying special attention to the mutual distances of particular motifs. In our previous research, we suggested that the CTCT motif acting as an EMRE (Lin et al., 1997) does not seem to be responsible for the activation of gene expression upon EMF exposure. The *in silico* analysis of ca. 30,000 potential promoter sequences in *A. thaliana* and ca. 40,000 in *S. tuberosum* showed approximately 130,000 and 150,000 CTCT motifs (within 1,000 nt upstream of genes), respectively, which would suggest that this element alone could not be responsible for the precise regulation of gene expression in response to the EMF (or possibly any other factor) and support the hypothesis that additional (or quite different) elements should assist in changing gene expression in response to EMF. Based on the transcriptome data, we aimed to find the actual EMRE motifs in plants by looking for the sequence motifs of the promoters of genes up- and downregulated after ELF-EMF exposure that repeat the most. Such motifs can be then used to create an artificial promoter whose sensitivity to ELF-EMF can be studied together with some reporter genes, like GUS (encoding β-glucuronidase).

## Data Availability

The datasets presented in this study can be found in onlinerepositories. The names of the repository/repositories and accessionnumber(s) can be found at https://www.ncbi.nlm.nih.gov/bioproject/?term=PRJNA516160.

## Abbreviations

4CL 4-Coumarate-CoA ligase; ANS, Anthocyanidin synthase; APx, Ascorbate peroxidase; BSMT, Benzoic acid/salicylic acid methyltransferase; C4H, Cinnamate-4-hydroxylase; CAD, Cinnamyl alcohol dehydrogenase; CAT, Catalase; CCR, Cinnamoyl-CoA reductase; CHS, Chalcone synthase; DEG, Differentially expressed gene; DFR, Dihydroflavonol reductase; F3′5′H, Flavonoid 3′,5′-hydroxylase; F3′H, Flavonoid 3′-hydroxylase; hae, Hours after exposure; PAL, Phenylalanine ammonia lyase; PR, Pathogenesis-related protein; ROS, Reactive oxygen species; SA, Salicylic acid; SAGT, Salicylic acid glucosyltransferase; SOD, Superoxide dismutase; TF, Transcription factor.
