# Erratum: Transcriptome profiling of flax plants exposed to a low-frequency alternating electromagnetic field

**DOI:** 10.3389/fgene.2023.1249870

**Published:** 2023-07-06

**Authors:** Frontiers Production Office

**Affiliations:** Frontiers Media SA, Lausanne, Switzerland

**Keywords:** electromagnetic field, flax, antioxidants, stress, transcriptome, RNA-seq

Due to a production error, there was an error in the author affiliations as published. During the production process, affiliations 2 and 3 were conflated, resulting in the following erroneous affiliation list being published:

Kamil Kostyn^1,^*, Aleksandra Boba^1,2^, Bartosz Kozak^1^, Dariusz Sztafrowski^3^, Jan Widuła^2^, Jan Szopa^1,2^ and Marta Preisner^1^

^1^Department of Genetics, Plant Breeding & Seed Production, Faculty of Life Sciences and Technology, Wroclaw University of Environmental and Life Sciences, Wroclaw, Poland

^2^Faculty of Electrical Engineering, Wroclaw University of Science and Technology, Wroclaw, Poland

^3^Faculty of Biotechnology, University of Wroclaw, Wroclaw, Poland

The corrected author affiliation information appears below:

“Kamil Kostyn^1,^*, Aleksandra Boba^1,3^, Bartosz Kozak^1^, Dariusz Sztafrowski^2^, Jan Widuła^3^, Jan Szopa^1,3^ and Marta Preisner^1^

^1^Department of Genetics, Plant Breeding & Seed Production, Faculty of Life Sciences and Technology, Wroclaw University of Environmental and Life Sciences, Wroclaw, Poland

^2^Faculty of Electrical Engineering, Wroclaw University of Science and Technology, Wroclaw, Poland

^3^Faculty of Biotechnology, University of Wroclaw, Wroclaw, Poland”

The publisher apologizes for this mistake. The original version of this article has been updated.

