# Supplementary figures and images for: Transcriptome profiling of flax plants exposed to a low-frequency alternating electromagnetic field
Source: Front Genet. 2023 Jun 7;14:1205469. doi: 10.3389/fgene.2023.1205469 (PMC10282948; doi:10.3389/fgene.2023.1205469)

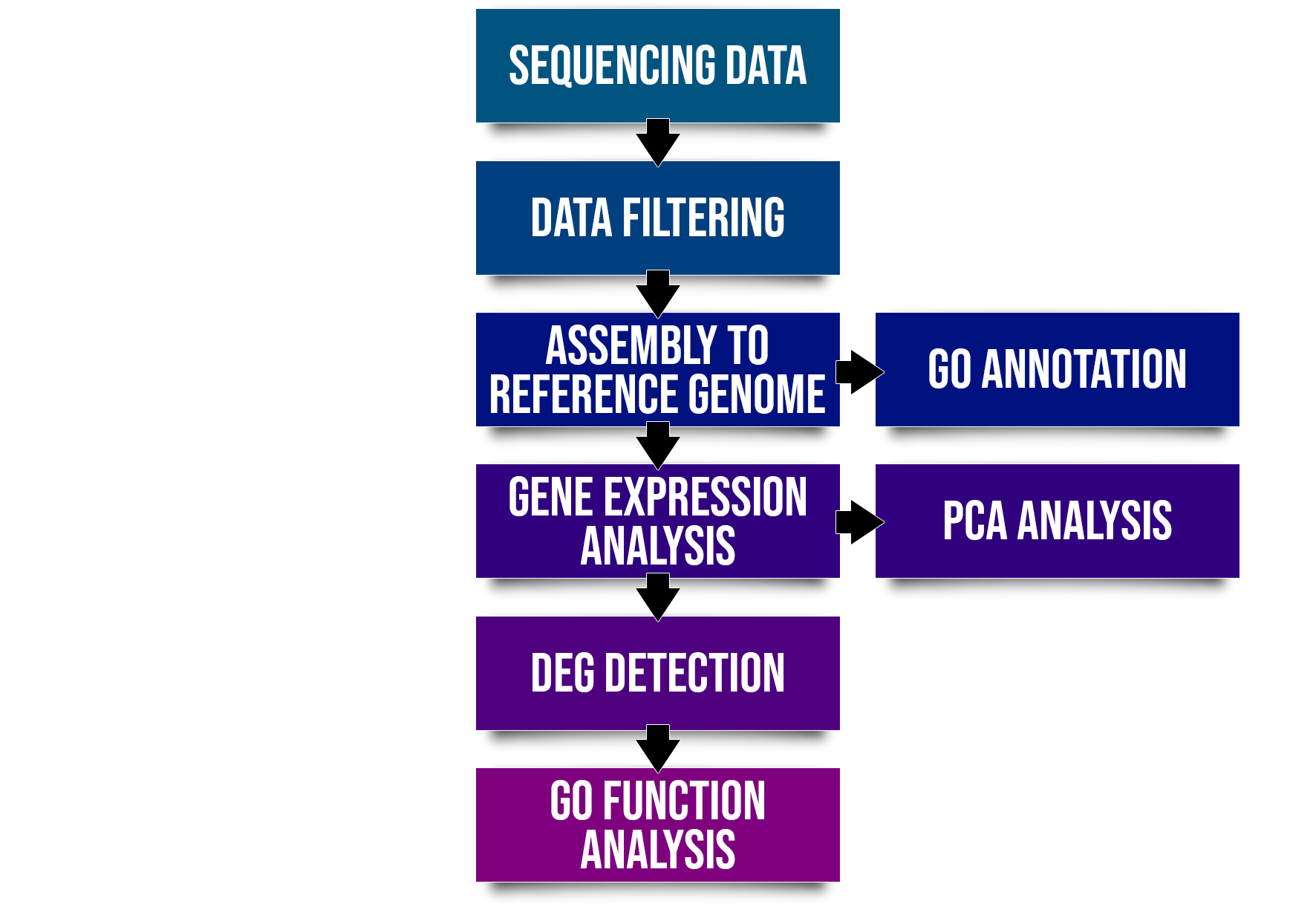

Supplement: Supplementary file 1 [file DataSheet1.zip › Supplementary Fig S1.jpg]

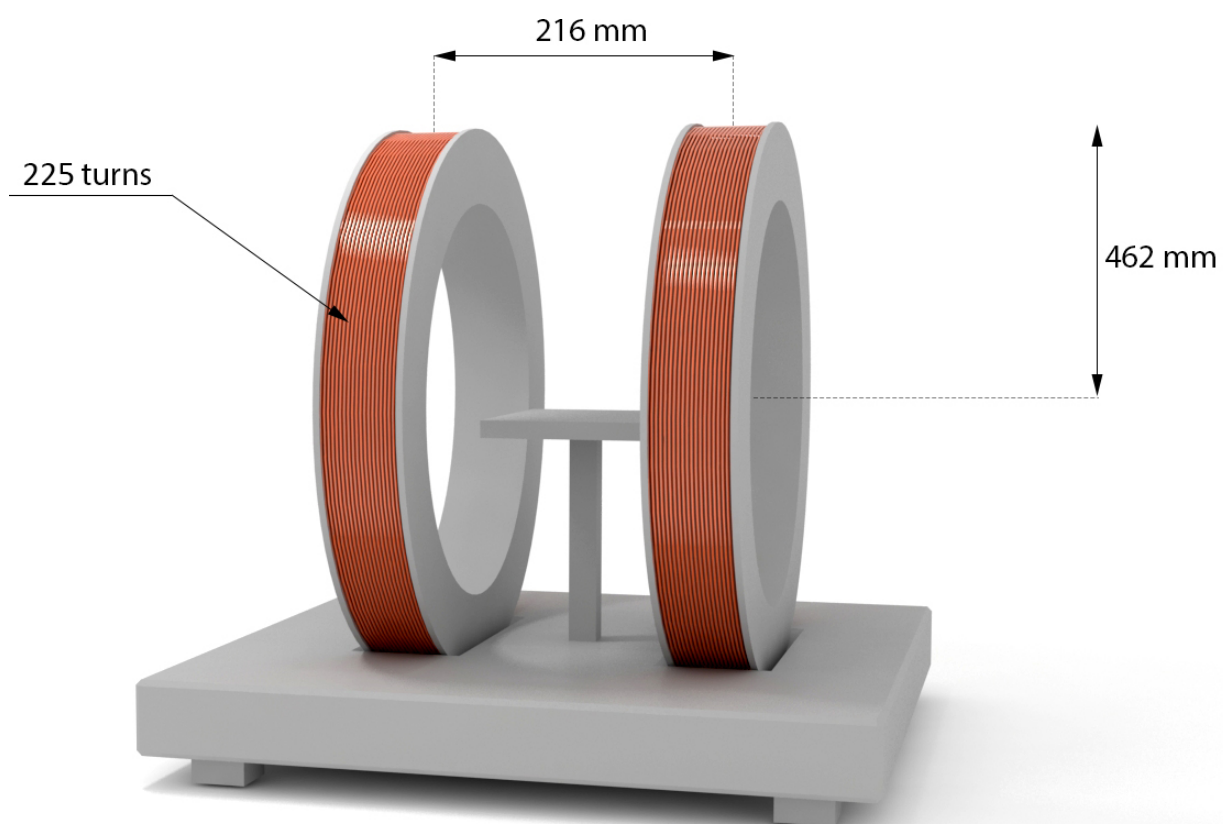

Supplementary Fig S2. Scheme of the ELF-EMF exposure system.

Supplement: Supplementary file 1 [file DataSheet1.zip › Supplementary Fig S2.pdf]
